# Cognitive Effects of Prenatal Exposure to SSRIs in Children: A Systematic Review and Meta‐Analysis

**DOI:** 10.1155/ijpe/7930070

**Published:** 2026-08-10

**Authors:** Fatima Shafiya Ali, Huda Fatima, Zainab Jasim Alqurain, Aroob Abdullah Takruni, Sana Hashim, Aya Sami Dahroug, Husna Irfan Thalib, Shyma Haidar, Mable Pereira

**Affiliations:** ^1^ General Medicine Practice, Batterjee Medical College, Jeddah, Saudi Arabia, bmc.edu.sa; ^2^ School of Medicine, Lincoln American University, Georgetown, Guyana

## Abstract

**Background:**

Depression and anxiety occur in about 10% of pregnancies. Selective serotonin reuptake inhibitors (SSRIs) are often prescribed, but their potential effects on fetal neurodevelopment remain uncertain. Because serotonin influences brain maturation, prenatal SSRI exposure may affect later cognition, language, or motor function.

**Methods:**

This systematic review and meta‐analysis evaluated whether prenatal SSRI exposure is associated with differences in child cognitive, language, or psychomotor development. PubMed and Scopus were searched through June 2025 for cohort, case‐control, or randomized studies reporting standardized neurodevelopmental outcomes in children (0–18 years) with documented in utero SSRI exposure compared with unexposed controls. Two reviewers independently screened studies, extracted data, and assessed bias (Newcastle–Ottawa Scale; RoB‐2). Random‐effects meta‐analyses estimated standardized mean differences (SMDs) with 95% confidence intervals (CIs).

**Results:**

Of 250 records identified, 17 studies met inclusion criteria, and 9 contributed to meta‐analysis. For general cognition, pooled SMD = −0.20 (95% CI −0.50 to 0.10) showed no significant difference between groups. Sensitivity analyses restricted to SSRI‐only cohorts and Bayley‐specific instruments yielded similar findings. Secondary outcomes also showed no differences: receptive language (SMD = 0.04; 95% CI −0.16 to 0.23), expressive language (SMD = −0.17; 95% CI −0.40 to 0.06), and psychomotor development (SMD = −0.18; 95% CI −0.67 to 0.31).

**Conclusions:**

Current evidence does not support a clinically meaningful impact of prenatal SSRI exposure on early cognitive, language, or psychomotor development. Observed effects were small, imprecise, and diminished when analyses were limited to SSRI‐only cohorts and harmonized outcome measures.

## 1. Introduction

Depression and anxiety affect an alarming 1 in 10 pregnancies, and selective serotonin reuptake inhibitors (SSRIs) are the most prescribed pharmaceuticals during pregnancy [1]. Untreated perinatal depression is associated with adverse maternal and child outcomes such as lack of self‐care, premature birth, and impaired maternal functioning. Thereby, clinicians must balance the benefits of treatment against potential fetal risks [2, 3]. At the same time, medication use in pregnancy raises concerns about neurodevelopment, particularly cognition, language, and motor skills in childhood.

Serotonin, specifically 5‐hydroxytryptamine (5‐HT), is central to mood regulation and also plays key roles in fetal brain development such as neuronal proliferation/migration, synaptogenesis, and cortical circuit formation, as well as broader processes (e.g., sleep, attention, memory, and stress responsivity via the HPA axis) [4]. SSRIs are widely known to cross the placenta and fetal blood–brain barrier, thus increasing extracellular 5‐HT and potentially altering serotonergic signaling during sensitive developmental windows, which may influence later cognitive and behavioral outcomes [5, 6].

Recent observational studies and a few randomized analyses have examined children exposed in utero to SSRIs. Their findings are mixed; some report small deficits in general cognitive or language scores, whereas others find no difference compared with unexposed peers. Interpretation is complicated by confounding by indication, comedications, timing or dose of exposure, prematurity, and other socioeconomic factors. [6] Heterogeneity in outcome instruments (e.g., Bayley Scales of Infant Development, McCarthy General Cognitive Index, WPPSI, and NEPSY) and assessment ages further limits comparability.

Prior syntheses have seldom pooled disparate neurodevelopmental domains or combined SSRI with other antidepressant classes, although many did not separate general cognition from language or psychomotor outcomes [7]. Even fewer reviews have systematically explored instrument‐specific effects or conducted sensitivity analyses that address mixed or non‐SSRI exposures, timing, or measurement differences. A focused, up‐to‐date quantitative synthesis of cognitive outcomes after prenatal SSRI exposure with parallel analyses for receptive/expressive language and psychomotor development is therefore needed to inform prescribing and counseling.

We aimed to conduct a systematic review and meta‐analysis to evaluate whether prenatal exposure to SSRIs is associated with differences in child cognitive development compared with no SSRI exposure. This review aimed to provide precise, clinically relevant estimates for families and clinicians weighing SSRI treatment during pregnancy against potential neurodevelopmental risks.

## 2. Methodology

### 2.1. Study Design

This review was carried out in accordance with the PRISMA guidelines and was registered with the international prospective register of systematic reviews (PROSPERO ID: CRD420251135540). A quantitative approach was considered appropriate because the included studies reported standardized test scores, which enabled statistical comparison across populations. By combining these data, the review was able to examine variation across study designs and populations and provide an accurate estimate of the relationship between prenatal SSRI exposure and child cognitive outcome. This approach was selected over a qualitative synthesis primarily because it provides objectivity, consistency, and the ability to identify minor changes in neurodevelopmental performance.

### 2.2. Eligibility Criteria

Research publications such as prospective or retrospective cohort studies, case‐control studies, or randomized controlled trials (RCTs), and studies published in English up to June 2025, were considered eligible. Children aged 0–18 years with documented prenatal exposure to SSRIs were compared with either (1) children born to mothers without depression and without SSRI exposure, or (2) children born to mothers with untreated maternal depression during pregnancy. Only studies that documented cognitive neurodevelopmental outcomes, such as IQ, memory, executive function, attention, language development, or standardized cognition test scores (e.g., Bayley Scales, WISC, and WPPSI), were included.

### 2.3. Exclusion Criteria

A comparison group consisted of children not exposed to an SSRI in utero. Excluded studies included those involving children outside the 0–18‐year age range, those published in languages other than English, and those that did not report neurodevelopmental outcomes relevant to cognition, language, or psychomotor development. Case reports, cross‐sectional studies, narrative or systematic reviews, meta‐analyses, scoping reviews, editorials or opinion articles, and research examining prenatal exposure to multiple psychotropic medications or unspecified antidepressants were all excluded.

### 2.4. Search Strategy

A complete literature search was performed using PubMed and Scopus. The PubMed search strategy included MeSH terms and keywords, such as “Selective Serotonin Reuptake Inhibitors” (“SSRI” or “individual drug names”), “Prenatal Exposure” (“in utero exposure” or “maternal drug use”), “psychomotor development” (“neurodevelopment” or “child development”), and “Child” (“infant” or “adolescent” or “pediatric”), with analogous keyword combinations applied across both databases.

### 2.5. Screening and Data Extraction

Two reviewers independently screened all titles, abstracts, and full texts for eligibility criteria, and disagreements were resolved by discussion with a third reviewer. Rayyan software was used to facilitate the screening process. Two reviewers extracted data independently on a Google Doc that included study design, country, sample characteristics, timing, and type of SSRI exposure, dosage, comparator group information, outcome measures, and follow‐up duration.

### 2.6. Risk Of Bias Assessment

The Cochrane RoB 2 tool was used to assess risk of bias in RCTs, whereas the Newcastle–Ottawa Scale was used in observational studies.

### 2.7. Data Synthesis and Statistical Analysis

A random‐effects model was used for meta‐analyses whenever feasible. For continuous outcomes, we estimated standardized mean differences (SMDs) with 95% confidence intervals (CIs) and used the *I* ^2^ statistic to determine the heterogeneity. Sensitivity analyses were prepared to assess the robustness of the findings. These included limiting analyses to SSRI‐only exposures (excluding mixed antidepressant use) and trials that only recorded Bayley scores. Studies that did not match prespecified methodological requirements (e.g., no compatible continuous outcomes or exposure limited to SSRIs) were eliminated from the quantitative synthesis but kept for qualitative analysis.

## 3. Results

**Figure 1:**
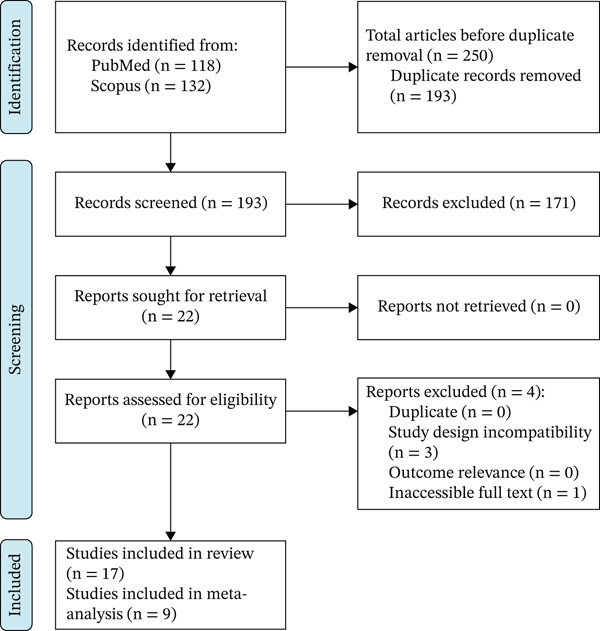
PRISMA flow diagram of study identification and screening.

We identified 250 records from PubMed (n = 118) and Scopus (n = 132). After removing 193 duplicates, 57 unique records remained for title/abstract screening; 35 were excluded as not relevant to the research question. We assessed 22 full‐text articles for eligibility and excluded 4 (3 due to incompatible study design, and 1 due to inaccessible full text). Seventeen studies met the inclusion criteria and were included in the systematic review, of which nine provided sufficient data for meta‐analysis (Figure 1).

### 3.1. Characteristics of Included Studies

This review included a diverse set of cohort and case‐control studies between the late 1980s and early 2020s, carried out in the United States, Canada, Norway, and Australia. The study designs covered were mainly longitudinal or prospective, whereas retrospective analyses were also considered. Sample sizes differed from large datasets of more than 200 mother–child pairs to small single‐site cohorts of 55–60 participants. Developmental assessments were conducted as early as birth and as late as 86 months of age, with periods of follow‐up extended from infancy to early school age.

Most mothers at the time of pregnancy were in their late 20s to mid‐30s across studies. Although reported inconsistently, maternal characteristics such as socioeconomic status, marital or partner status, and parity were frequently used to adjust analyses. Standard measurements such as Beck depression inventory (BDI), Edinburgh postnatal depression scale (EPDS), CES‐D, and Hamilton depression rating scale were mainly used to assess anxiety and depression, the two main psychiatric diagnoses evaluated. Maternal self‐report, medical records, or prescription databases were used to measure exposure to SSRIs. The most examined medications were fluoxetine, paroxetine, sertraline, citalopram, and escitalopram (Table 1). Multiple studies made a distinction between the first, second, or third trimester, and continued consumption throughout the pregnancy, although the timing of exposure varied. Dosage information was often categorized into wide low, middle, and high ranges by some studies, such as Casper, and in others, it was rarely reported. A few also provided drug‐specific values such as fluoxetine 28–80 mg/day (Nulman et al. [11]), sertraline about 11 mg on average (Casper et al. [13]), and paroxetine 20–25 mg, fluoxetine 20 mg, and escitalopram 10 mg (Batton et al. [14]) (Table 2).

**Table 1:** General characteristics of the maternal participants.

First author′s last name	Year	Country	Study design	Sample size	SSRI‐exposed	Control	Duration of follow‐up	Time frame	Maternal age	Parity	Marital status	Socioeconomic status (SES)	Mental health score	Treatment
van der Veere [8]	2020	The Netherlands	Prospective cohort	102	61	41	2.5 years	2009–2012	24–42 years	Not reported	Not reported	Low ed: 45% (SSRI), 38% (control)	BDI median 7 vs. 4; STAI median 40 vs. 27.5	Paroxetine, citalopram, fluoxetine, etc.
Casper [9]	2011	United States	Cohort study	55	55	Not reported	Up to 40 months; mean 14 months	Not reported	34.1–4.0 years	1.6–2.1	Not reported	Not reported	BDI = 1st tri = 25.3 (1.1), 2nd/3rd tri = 25.6 (3.5), con expo = 19.6 (5.5), HMD CES‐D	Sertraline, fluoxetine, paroxetine, and citalopram
Austin [10]	2013	Australia	Longitudinal cohort study	58	35	23	17–24 months	2007–2010	34.9–32.7 years	Not reported	Not partnered = 2; partnered = 33	No university = 9 (26); university/postgraduate = 26 (74),	EPDS at baseline: 7.4 (5.5) and at follow‐up: 6.2 (4.9)	Sertraline, fluoxetine, citalopram, escitalopram, paroxetine, fluvoxamine; venlafaxine, and dothiepin
Nulman [11]	2002	Canada	Controlled cohort study	135	Fluoxetin = 40	36 (psychiatric), 10 (healthy)	Not reported	Not reported	Fluoxetine 32.2–30.7 years	1.1–0.7	Not reported	Hollingshead index of SES 44.8 (12.7)	(CES‐D Scale) 39.9 (11.3)	Fluoxetine
Hanley [12]	2013	Canada	Retrospective cohort study	83	31	52	36 weeks–10 months	2000–2007	33.5–34.6 years	Not reported	Not reported	(1) high school or less = (0), postgraduate = 9 (29.0)	HAMD**s** **c** **o** **r** **e** **s** = 10.6 (6.7) vs. 11.1 (4.7) vs. 10.1 (7.4), and EPDS *s* *c* *o* *r* *e* *s* = 6.2 (4.2) vs. 6.8 (4.2) vs. 6.6 (5.0)	Paroxetine, fluoxetine, sertraline, and venlafaxine and citalopram
Casper [13]	2003	United States	Case‐control study	Not reported	31	13	Not reported	1998–2000	34.9–36.6 years	1.65–1.62	90%	Edu‐16.8 (2.8)	BDI; 21.3 (7.9)	Fluoxetine, paroxetine, and sertraline
Batton [14]	2012	United States	Retrospective case control study	Not reported	19	19	6, 12, 24, and 36 months	2001–2008	28–27 years	Singleton	Not reported	18 (95%) mothers graduated from high school, and 9 (47%) had a college degree.	Not reported	Paroxetine, fluoxetine, sertraline, citalopram, and escitalopram oxalate
Nulman[15]	1997	Canada	Cohort study	219	55	84	86 months after birth	1988–unknown	31–30 years	1–0.4	Not reported	Hollingshead Four Factor Index: 40 ± 13 (lower socioeconomic status),	CES Depressed Mood Scale: score on best days, 13 ± 10; score on worst days: 35 ± 15	Fluoxetine
Cohen [16]	2022	United States	Cohort study	54	18	18	Not reported	1999–2007	35.9 ± 4.6	Not reported	Not reported	Some college or associate′s degree 2 ± 4.6; bachelor′s degree‐12 ± 27.3; master′s degree‐20 ± 45.5; doctoral degree‐10 ± 22.7	Not reported	SSRI
Hermansen [17]	2016	Norway	Cohort study	103	28	33	Not reported	1998–2008	29–32 years	Not reported	In relationship: 23 ± 85.20; single 4 ± 14.80.	3.24 ± 1.22	Not reported	SSRI

**Table 2:** General characteristics of the paediatric participants.

First author′s last name	Year	Age at assessment	Sex distribution—M/F	Gestational age at birth	Birth weight	Height at testing (percentile)	Fronto‐occipital circumference at testing	Dosage
van der Veere [8]	2020	30.1 months (range 28.1–35.2)	46/56	39 weeks	3438 ± 588 vs. 3805 ± 478 g	Not reported	Not reported	Not reported (varied)
Casper [9]	2011	12–40 months (mean 14 months)	Not specified	39 ± 2 wks (SSRI) vs. 40 ± 1 wks (control)	3567 ± 683 g (SSRI) vs. 3373 ± 540 g (control)	47 ± 27 (SSRI) vs. 49 ± 31 (control)	45 ± 28 (SSRI) vs. 48 ± 27 (control)	Average dose categorized as low/medium/high
Austin [10]	2013	19.1 months (SSRI), 18.9 (control)	SSRI:16/19, control: 9/14	39.3 (1.1) (SSRI), 39.7 (1.5) (control) (weeks)	3586 (543) (SSRI), 3585 (583) (control)	52.0 ± 2.7	34.5 ± 1.6, 35.0 ± 1.4	(1st vs. 2nd vs 3rd tri) sertraline 57.5 vs. 60 vs. 62.5, fluoxetine 24 vs. 25 vs. 25, citalopram 18 vs. 22 vs. 18, escitalopram 5 vs. 5 vs. 5, paroxetine 20 vs. 20 vs. 20, fluvoxamine 100 vs. 100 vs. 100, venlafaxine 150 vs. 168 vs. 178, and dothiepin 20 vs. 35 vs. 60
Nulman [11]	2002	15–71 months (psychometrist), 15–30 months (BSID III)	Not reported	39.4 weeks (no difference among groups)	Mean ≈ 3400 g (range 3403–3531 g; no sig. difference)	SSRI: 51.3 ± 30.1, control: 62.6 ± 26.3	SSRI: 43.7 ± 26.1, control: 54.1 ± 23.0	Fluoxetine: (28–80 mg a day)
Hanley [12]	2013	At 36 weeks and 10 months	SSRI: 14/20, control: 28/29	SSRI: 38.8 (1.5), control: 39 (2.1) weeks	SSRI: 3292.7 g(515.2), control: 3435.2 (555.3)	E = 50.6 (2.13), c = 51.6 (2.76)	Not reported	Not reported
Casper [13]	2003	Birth	Not reported	SSRI = 39.1 (1.1), control = 38.7 (1.5)	SSRI = 3394 (432.2), control = 3363 (498.5)	E = 50.3 ± 2.5, c = 49.7 ± 7.2	E = 54.2 ± 25.9, c = 50.3 ± 28.1	Sertraline = 11.9 ± 9.5 months/119.4 ± 75.8 mg, paroxetine = 7.8 ± 7.3 months/17.2 ± 10.1 mg, and fluoxetine = 3 ± 1.7 months/
Batton [14]	2012	24–36 months	Not reported	SSRI exposed: 28 ± 2.9 weeks; control: 28 ± 2.7 weeks	SSRI exposed: 1018 ± 367 g; control: 1044 ± 385 g	Not reported	Not reported	Paroxetine (20, 25 mg), fluoxetine (20 mg), sertraline (50 mg), citalopram (20 mg), and escitalopram oxalate (10 mg)
Nulman [15]	1997	16‐86 months	Not reported	SSRI: 39 ± 2, control: 40 ± 1	SSRI: 3567 ± 683, control: 3373 ± 540	SSRI: 47 ± 27, control: 49 ± 31	SSRI: 45 ± 28, control: 48 ± 27	Fluoxetine (n = 55)
Cohen [16]	2022	12.6 ± 2.6	M not reported/18	Mean: 38.4, SD: 2.4	Not reported	Not reported	Not reported	Not reported
Hermansen [17]	2016	Pre‐ aged school children	M not reported/SSRI: 16, control: 18	SSRI: 39.17 ± 1.78, control: 39.55 ± 1.97	SSRI: 3482.78 ± 380.44, control: 3474.12 ± 413.17	Not reported	Not reported	Not reported

Children born to healthy mothers and those who had untreated depression were both included as a control group, to allow differentiation between the influence of maternal mood disorders and pharmaceutical effects. According to studies, many infants were delivered at term, with average gestational ages ranging between 38 and 40 weeks. Birth weights typically ranged between 3200 and 3600 g, which falls within the normal range and did not differ significantly between the exposed and control groups, whereas a few preterm samples documented weights lower than 1000 g. Sex distribution was balanced across the studies; however, some reported a slightly higher proportion of females in the SSRI‐exposed group. With testing ages ranging from 6 months to nearly 7 years, follow‐up assessments included developmental stages from infancy through early childhood (Table 2).

The provided extensive assessments of prenatal SSRI exposure, maternal mental health, and child neurodevelopment, despite variability in population size, methodology, and follow‐up length, provide an extensive overview of early verbal, behavioral, and cognitive effects in children exposed to SSRI. Collectively, these studies present a comprehensive picture of early cognitive behavioral and language outcomes in exposed children.

### 3.2. General Cognition

**Figure 2:**
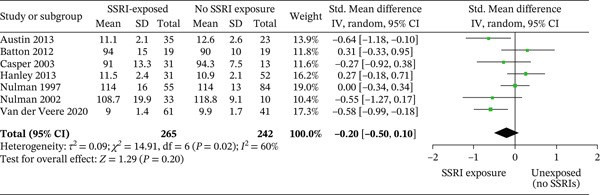
Comparison of general cognitive performance between children with prenatal SSRI exposure and nonexposed controls.

Seven studies (SSRI‐exposed *n* = 265; unexposed *n* = 242) reported continuous general‐cognition scores using different instruments. A random‐effects model (SMD) showed no clear difference between children with prenatal SSRI exposure and unexposed controls: SMD −0.20 (95% CI −0.50 to 0.10); test for overall effect *Z* = 1.29, *p* = 0.20. Heterogeneity was moderate (*τ* ^2^ = 0.09; *χ* ^2^ ≈ 14.9 on 6 df; *I* ^2^ ≈ 60*%*) (Figure 2).

### 3.3. Mental Development

**Figure 3:**
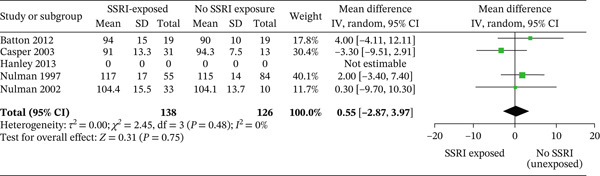
Comparison of mental development between children with prenatal SSRI exposure and nonexposed controls.

Five studies reported Bayley Mental Development Index (MDI) and were pooled using a random‐effects model. There was no clear difference in mental development between children with prenatal SSRI exposure and unexposed controls (MD 0.55 points, 95% CI −2.87 to 3.97; *τ* ^2^ = 0.00; *χ* ^2^ = 2.45, df = 3, *p* = 0.48; *I* ^2^ = 0*%*; test for overall effect: *Z* = 0.31, *p* = 0.75) (Figure 3).

### 3.4. Communication (Receptive and Expressive Language Outcomes)

We analyzed receptive and expressive language as separate outcomes because they are distinct domains measured by separate subscales on the included instruments (Bayley Language: receptive/expressive scaled scores, Reynell Verbal Comprehension/Expressive Language).

**Figure 4:**
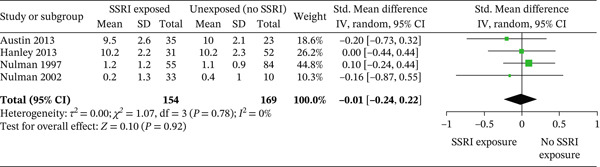
Comparison of receptive language expression between children with prenatal SSRI exposure and nonexposed controls.

Four observational studies contributed data: two studies using Bayley‐III receptive language scaled scores [10, 12] and two using the Reynell Verbal Comprehension scale [11, 15]. Because instruments differed, we pooled SMDs (random‐effects). Across 154 SSRI‐exposed and 169 unexposed children, there was no clear evidence of a difference in receptive language: SMD −0.01 (95% CI −0.24 to 0.22). Statistical heterogeneity was absent (*τ* ^2^ = 0.00; *χ* ^2^ = 1.07, df = 3, *p* = 0.78; *I* ^2^ = 0*%*) (Figure 4).

**Figure 5:**
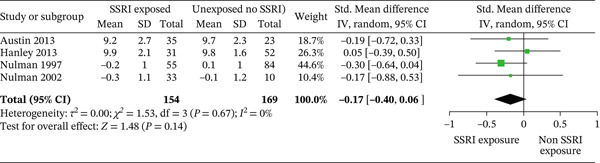
Comparison of expressive language expression between children with prenatal SSRI exposure and nonexposed controls.

Four studies contributed expressive language data [10–12, 15], using Bayley Expressive Language scaled scores [10, 12] and the Reynell Expressive Language scale [11, 15]. Because instruments differed, we pooled SMDs. There was no clear difference in expressive language between children with prenatal SSRI exposure and unexposed controls (SMD −0.17, 95% CI −0.40 to 0.06; *τ* ^2^ = 0.00; *χ* ^2^ = 1.53, df = 3, *p* = 0.67; *I* ^2^ = 0*%*; test for overall effect *Z* = 1.48, *p* = 0.14; SSRI *n* = 154, unexposed *n* = 169). The point estimate slightly favored the unexposed group, but the CI included no effect (Figure 5).

### 3.5. Psychomotor Development

**Figure 6:**
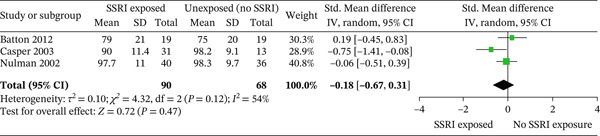
Comparison of psychomotor development expression between children with prenatal SSRI exposure and nonexposed controls.

Three studies (SSRI‐exposed *n* = 90; unexposed *n* = 68) reported psychomotor outcomes on different scales and were pooled using SMD (random‐effects, inverse‐variance). There was little to no difference between infants prenatally exposed to SSRIs and unexposed controls (SMD −0.18, 95% CI −0.67 to 0.31; *Z* = 0.72, *p* = 0.47. Between‐study heterogeneity was moderate (*τ* ^2^ = 0.10; *χ* ^2^ = 4.32, df = 2, *p* = 0.12; *I* ^2^ = 54*%*). Individual study effects ranged from a small benefit for exposure (Batton 2012: SMD 0.19 [−0.45, 0.83]) to a moderate detriment (Casper 2003: SMD −0.75 [−1.41, −0.08]), with one study showing no clear difference (Nulman 2002: SMD −0.06 [−0.51, 0.39]) (Figure 6).

### 3.6. Risk of Bias Assessment

**Figure 7:**
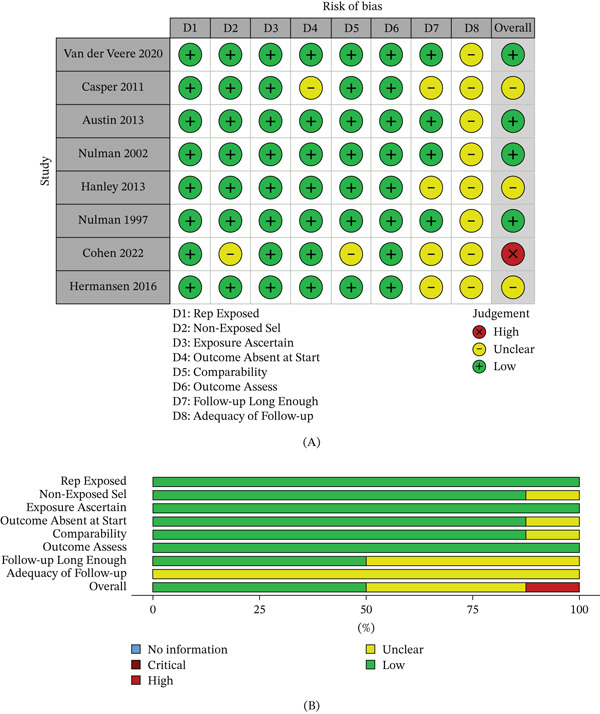
(A) Risk‐of‐bias traffic‐light plot for nonrandomized studies assessed with the Newcastle–Ottawa Scale across eight domains. (B) Summary stacked bar plot showing the distribution of judgments (low, unclear, high) across all included nonrandomized studies.

**Figure 8:**
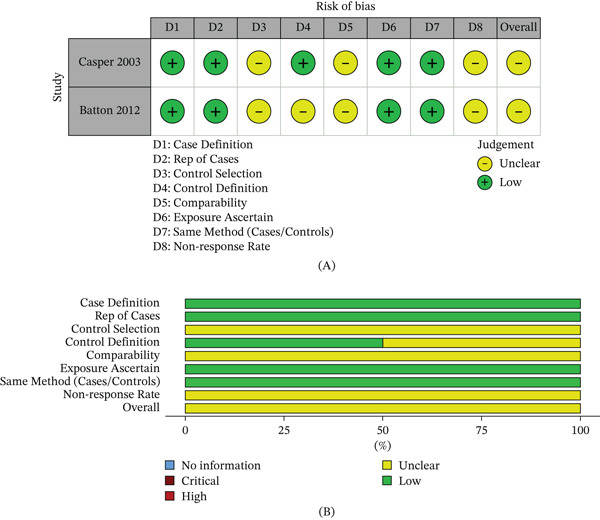
(A) Risk‐of‐bias traffic‐light plot for the case‐control studies (i.e., Casper 2003 and Batton 2012) [13, 14] appraised with the Newcastle–Ottawa Scale across eight domains. (B) Domain‐level summary plot showing the distribution of judgments (low vs. unclear) across the case‐control studies; no high‐risk ratings were assigned, with most uncertainty in the control definition/selection domains.

Using the Newcastle–Ottawa Scale, most cohort studies were good quality and low risk typically 7–8, with clear exposure definition, standardized outcome assessment, and similar methods across groups [8, 10, 11, 15] (Figure 7). A few cohorts were moderate due to incomplete adjustment for confounders or short or uneven follow‐up Hanley 2013 and Hermansen 2016, and one Cohen 2022 [12, 16, 17] was judged higher risk because of selection and attrition concerns. The two case‐control studies in Casper 2003 and Batton 2012 [13, 14] were moderate or unclear risk, mainly from control selection and comparability domains, despite adequate exposure and outcome ascertainment (Figure 8). Overall, the evidence base is methodologically acceptable for synthesis and domain‐level limitations (especially confounding and follow‐up) were considered in interpretation and explored in sensitivity analyses.

## 4. Discussion

Across observational cohorts and small trials, prenatal exposure to SSRIs was not associated with a clinically important decrement in early childhood cognition. In the primary analysis of general cognition, pooled effects were small and imprecise and centered near the null value. When we restricted analyses to the Bayley Cognitive/MDI only (in order to align instruments) and SSRI‐only exposure (thereby minimizing exposure heterogeneity) [14], the direction and magnitude remained similar, and heterogeneity fell substantially. For specific domains, pooled estimates showed no clear differences in receptive language (SMD ≈ 0.04, 95% CI spanning the null), a small, nonsignificant reduction in expressive language (SMD ≈ −0.17), and no clear effect on psychomotor development (SMD ≈ −0.18). Overall, the data do not support a material adverse effect of prenatal SSRI exposure on early cognitive or psychomotor outcomes.

Any signal, when present, tended to be small and negative, that is, favoring the unexposed group) but statistically uncertain. Our pooled differences (whether SMDs near 0 or MDs ~0–1 point on Bayley) are unlikely to be clinically meaningful at the population level. These findings are consistent with the view that maternal illness severity and family‐level factors explain much of the crude difference observed between exposed and unexposed children [14, 16]. Studies that adjusted more completely for maternal mood during pregnancy and postpartum, socioeconomic variables, smoking/alcohol, and paternal factors tended to show attenuated associations. Importantly, untreated perinatal depression and anxiety themselves are associated with preterm birth, suboptimal maternal–child bonding, and developmental risk. Our results, therefore, do not support discontinuing an otherwise effective SSRI solely to prevent cognitive harms in the child, particularly when maternal disease control would be compromised [17].

We observed appreciable heterogeneity in the broad General Cognition outcome that diminished after harmonizing instruments (Bayley‐only scale) and excluding mixed antidepressant exposures. This suggests that measurement nonequivalence and exposure misclassification, such as mixed drugs or doses, were major sources of between‐study variability. Age at testing also differed (infancy to preschool), and cognitive scores are age‐sensitive and may not track linearly across tools.

We prespecified a narrow clinical question to cognitive effects only, extracted means and SDs to compute comparable effect sizes, and ran targeted sensitivity analyses (i.e, instrument‐restricted and SSRI‐only). We also separated language into receptive and expressive domains to avoid diluting potentially different effects.

Most included studies were observational, leaving room for residual and unmeasured confounding. Additionally, the literature search was limited to PubMed and Scopus because of feasibility and institutional access constraints. Although these databases provide broad biomedical coverage, the exclusion of databases such as Embase and PsycINFO may have resulted in the omission of potentially relevant studies. Exposure timing and dosage were also inconsistently reported, and only a few studies examined trimester‐specific or dose–response effects [11, 12, 15].

Another important limitation was the heterogeneity of comparator groups across studies, as some studies used children of mothers without depression, whereas others included children of mothers with untreated maternal depression as controls. Due to the limited number of studies and inconsistent outcome reporting, formal subgroup analyses based on comparator type could not be reliably performed, which may have contributed to residual confounding by indication. Outcome measures also varied substantially, and several studies reported only subscales or *p* values, leading to exclusion from quantitative synthesis.

Furthermore, risk‐of‐bias assessments frequently identified concerns related to selection bias, differences between exposed and unexposed families, and attrition [14]. Because fewer than 10 studies were available for most outcomes, publication bias and small‐study effects could not be robustly assessed. Finally, most neurodevelopmental outcomes were evaluated before school age, leaving the long‐term effects on academic performance and executive functioning uncertain [8, 13].

Future studies should use common, age‐standardized cognitive batteries (for instance, Bayley cognitive index at 12–24 months and comparable language tools) so results are directly combinable. Exposure must be described precisely (e.g., drug, dose, adherence, and trimester) so that drug‐ and dose‐specific effects can be tested. Because confounding is the main threat, designs that control it better—such as sibling‐comparison, negative‐control analyses, or causal methods with robust adjustment for maternal and paternal cognitive ability, illness severity, SES, and alcohol—are needed. Cohorts should follow children into school age to capture IQ, executive function, and academic outcomes, not just infant scores.

Across the best available evidence, prenatal SSRI exposure is not associated with a clinically meaningful detriment in early general cognition, language, or psychomotor development [9]. Any possible effects are small and uncertain and appear sensitive to measurement and confounding. Ensuring maternal mental health remains paramount and higher‐quality longitudinal research should clarify drug‐specific and trimester‐specific risks and track outcomes beyond early childhood [12].

## 5. Conclusion

This systematic review and meta‐analysis found no association between prenatal exposure to SSRIs and deficits in early childhood cognition, psychomotor development, and language. Pooled estimates were small, statistically nonsignificant, and largely centered around the null effect, even with some studies revealing a slight negative trend favoring unexposed children. The accuracy of these findings was validated by sensitivity analyses restricted to SSRI‐only populations and standardized measures. These findings support the ongoing administration of SSRIs during pregnancy when clinically appropriate, given the potential risk of untreated maternal depression and anxiety for both mother and child. However, to better understand the effects that are specific to a certain drug or trimester and to broaden the assessment of results in school‐age and beyond, further investigation using larger longitudinal cohorts and standardized neurodevelopmental markers is crucial.

This review contributes to the field by offering a focused and clinically relevant synthesis of cognitive, language, and psychomotor outcomes after prenatal SSRI exposure, rather than combining all neurodevelopmental outcomes into a single broad category. Its use of domain‐specific analyses and sensitivity analyses restricted to SSRI‐only exposure and standardized outcome measures strengthens the interpretability of the findings. By separating potential medication effects from methodological heterogeneity, this study provides clearer evidence to support counseling and shared decision‐making in perinatal mental health care.

## Funding

No funding was received for this manuscript.

## Ethics Statement

This study is a systematic review and meta‐analysis of previously published literature and did not involve direct human participation.

## Consent

Consent is not applicable because no human participants were directly involved, and all data were obtained from previously published studies.

## Conflicts of Interest

The authors declare no conflicts of interest.

## Data Availability

Data sharing is not applicable to this article as no datasets were generated or analyzed during the current study.
